# Prognostic impact of low BMI on outcomes following definitive radiotherapy for esophageal squamous cell carcinoma

**DOI:** 10.3389/fnut.2026.1774890

**Published:** 2026-06-17

**Authors:** Jian Wang, Junhui Wang, Jianxin Chen

**Affiliations:** 1 Department of Education, Department of International Ward, The Quzhou Affiliated Hospital of Wenzhou Medical University, Quzhou People’s Hospital, Quzhou, Zhejiang, China; 2 Department of Gastroenterology, Jiaxing Second Hospital, Jiaxing, Zhejiang, China; 3 Department of Radiation, The Quzhou Affiliated Hospital of Wenzhou Medical University, Quzhou People’s Hospital, Quzhou, Zhejiang, China; 4 School of Medicine, Shaoxing University, Shaoxing, Zhejiang, China

**Keywords:** body mass index, esophageal squamous cell carcinoma, radiotherapy, survival, treatment-related toxicity

## Abstract

**Background:**

Esophageal squamous cell carcinoma (ESCC) is a globally significant malignancy with a poor prognosis. While definitive radiotherapy is a primary curative-intent treatment for early-to-mid-stage ESCC, patient outcomes remain heterogeneous. Nutritional status, as reflected by body mass index (BMI), is increasingly recognized as a critical host-related factor influencing cancer prognosis. However, evidence regarding the impact of low BMI on survival and treatment-related toxicity in ESCC patients treated with radiotherapy remains limited.

**Methods:**

This retrospective, single-center cohort study analyzed 135 patients with stage I–III ESCC who received definitive radiotherapy between 2021 and 2024. Patients were stratified into low BMI (<18.5 kg/m^2^, *n* = 33) and non-low BMI (≥18.5 kg/m^2^, *n* = 102) groups based on WHO criteria. Baseline characteristics, laboratory parameters, and treatment details were collected. Survival outcomes (overall survival [OS] and progression-free survival [PFS]) were evaluated using Kaplan–Meier analysis and Cox regression models to identify independent prognostic factors. Treatment-related adverse events (AEs) were graded per CTCAE v5.0.

**Results:**

The low BMI group had a significantly higher proportion of advanced TNM stage III disease (78.8% vs. 49.0%, *p* = 0.011) and nutritional risk (NRS2002 ≥ 3: 93.9% vs. 72.5%, *p* = 0.010). With a median follow-up of 24.5 months, the low BMI group exhibited markedly inferior median OS (16.60 vs. 38.30 months, *p* = 0.010), though PFS did not differ significantly (11.60 vs. 19.80 months, *p* = 0.106), despite a numerical trend toward worse PFS in the low BMI group. Multivariate analysis identified nutritional risk (HR = 2.100, *p* = 0.047) as an independent predictor of OS. The low BMI group also showed numerically higher rates of hematological and non-hematological toxicities, including severe anemia (12.1% vs. 2.9%) and radiation esophagitis (75.7% vs. 50.0%).

**Conclusion:**

Low baseline body mass index (BMI) was associated with inferior unadjusted overall survival and a numerically higher burden of treatment-related toxicity in ESCC patients undergoing definitive radiotherapy. However, after accounting for TNM stage and NRS2002-defined nutritional risk, BMI did not remain an independent prognostic factor. These findings suggest that BMI should be interpreted as a simple surrogate marker of nutritional vulnerability and adverse baseline clinical status. Comprehensive nutritional assessment and early supportive intervention should be integrated into multidisciplinary management for this vulnerable population.

## Introduction

Esophageal carcinoma (EC) represents a significant global health challenge, characterized by aggressive tumor biology and a generally poor prognosis. It ranks as the seventh most commonly diagnosed cancer and the sixth leading cause of cancer-related mortality worldwide (1). Esophageal squamous cell carcinoma (ESCC) is the predominant histological subtype, particularly in Eastern Asia and Eastern Africa (1, 2). For patients with early-to-mid-stage, non-metastatic ESCC, definitive radiotherapy, often combined with concurrent chemotherapy, serves as a primary curative-intent treatment modality, especially for those who are not suitable candidates for surgical resection (3, 4). While advancements in radiation techniques, such as intensity-modulated radiotherapy (IMRT), have improved precision and potentially reduced toxicity, treatment outcomes remain heterogeneous, underscoring the critical need for reliable prognostic and predictive biomarkers to optimize individual patient management (5–8).

Beyond established clinical factors like TNM stage and performance status, there is growing recognition of the importance of host-related factors in determining cancer treatment outcomes. Among these, nutritional status has emerged as a key determinant (9). Body mass index (BMI) serves as a simple, widely available surrogate marker of overall nutritional status and body composition. The relationship between BMI and cancer prognosis often exhibits a U-shaped or J-shaped curve, wherein both obesity and undernutrition are associated with adverse outcomes, though the mechanisms likely differ (10). In the context of EC, low BMI is frequently indicative of cancer cachexia, a multifactorial syndrome characterized by ongoing loss of skeletal muscle mass (with or without loss of fat mass) that leads to progressive functional impairment (11). This syndrome is driven by a complex interplay of tumor-derived factors, systemic inflammation, and metabolic dysregulation, which can not only compromise a patient’s physiological reserve to withstand anticancer treatments but also potentially promote a more aggressive tumor microenvironment (12, 13).

The prognostic significance of low BMI in esophageal cancer patients undergoing surgical resection has been relatively well-documented, with numerous studies consistently linking pre-operative underweight status to increased postoperative complications and worse overall survival (14–16). However, the impact of low BMI specifically on outcomes in the cohort of ESCC patients treated with definitive radiotherapy is less comprehensively explored. Existing literature in this area is often limited by small sample sizes, inclusion of mixed histologies (adenocarcinoma and squamous cell carcinoma), or a primary focus on surgical cohorts, leaving a gap in evidence pertinent to the radiotherapy-treated population (17). Furthermore, the interaction between low BMI and treatment-related toxicity profiles in the setting of radical radiotherapy is not fully elucidated. Given that radiotherapy can induce significant local inflammation and side effects such as esophagitis and dysphagia, which can further exacerbate nutritional decline, patients with a low BMI at baseline may be particularly vulnerable to a cycle of worsening nutrition and treatment intolerance (18–20).

Therefore, the primary objective of this real-world study was to investigate the association between baseline BMI (dichotomized as < 18.5 kg/m^2^ vs. ≥ 18.5 kg/m^2^ according to WHO criteria) and survival outcomes as well as treatment-related adverse events in a well-defined cohort of patients with stage I–III ESCC who received definitive radiotherapy. We hypothesized that patients with a low BMI would experience significantly inferior overall survival and progression-free survival, and bear a higher burden of treatment-related toxicities compared to their non-low BMI counterparts. By leveraging detailed clinical data and robust statistical analyses, this study aims to provide valuable insights into the role of nutritional status as a critical factor in the multidisciplinary management of ESCC, potentially identifying a patient subgroup that may benefit from intensified nutritional support and closer monitoring during radical radiotherapy.

## Materials and methods

### Study design and patient population

**FIGURE 1:**
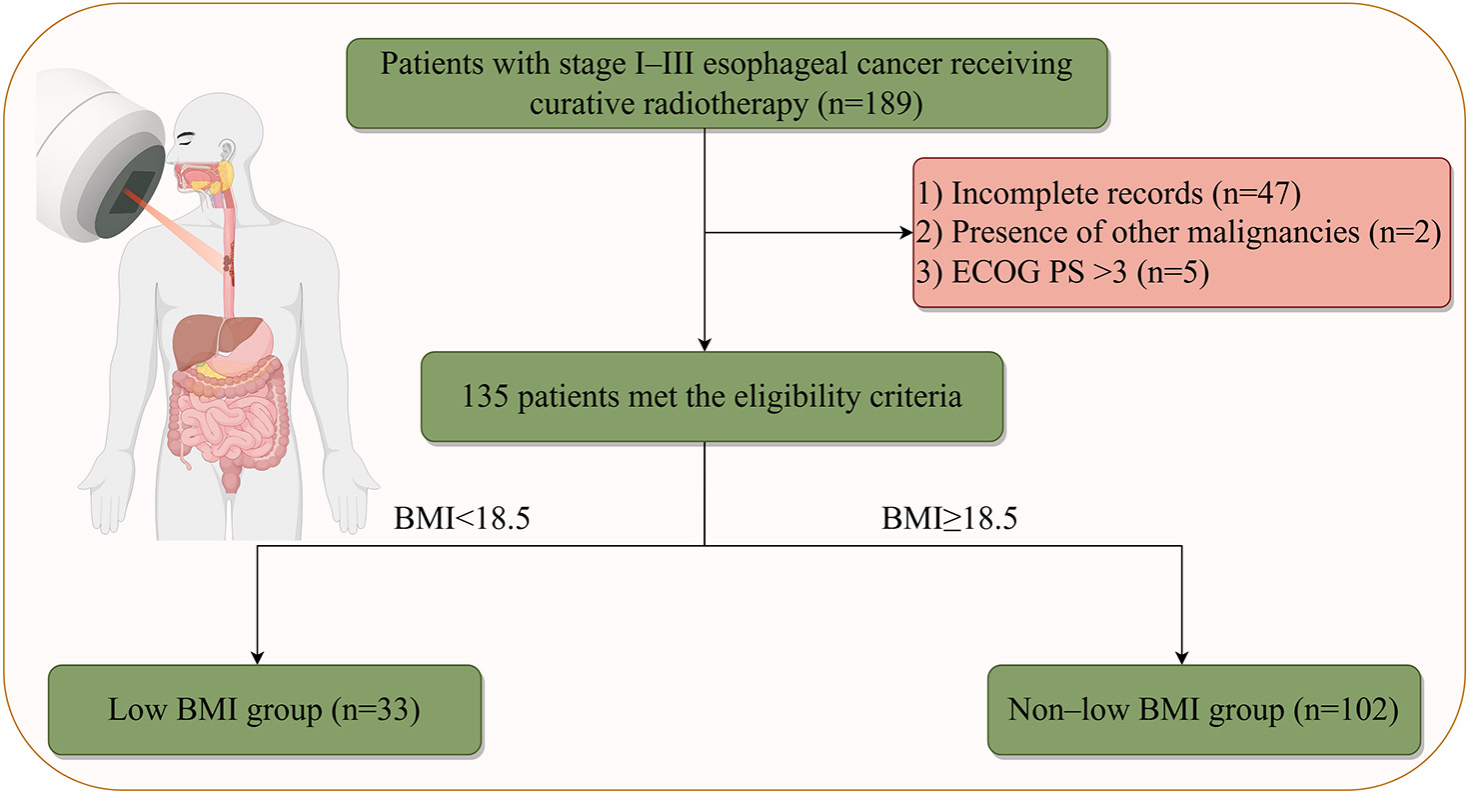
Patient enrollment flowchart. BMI, body mass index; ECOG PS, Eastern Cooperative Oncology Group Performance Status.

This retrospective, real-world cohort study was conducted at a single oncology center. The study protocol was reviewed and approved by the Institutional Review Board (IRB), which granted a waiver for informed consent due to the retrospective nature of the analysis. We systematically screened the electronic medical records of all patients diagnosed with histologically confirmed esophageal squamous cell carcinoma (ESCC) who received definitive radiotherapy at our institution between 1 January 2021, and 31 December 2024. The patient selection process is detailed in Figure 1.

### Inclusion and exclusion criteria

Patients were eligible for inclusion if they met all the following criteria: (1) aged 18 years or older; (2) newly diagnosed, non-metastatic (Stage I–III) ESCC confirmed by histology; (3) completed a full course of definitive radiotherapy with or without concurrent chemotherapy.

Key exclusion criteria were as follows: (1) incomplete medical records precluding accurate data extraction; (2) history of any other synchronous malignant tumor within 5 years prior to the ESCC diagnosis; (3) an Eastern Cooperative Oncology Group Performance Status (ECOG PS) greater than 3 at the initiation of radiotherapy; and (4) palliative intent of treatment.

For patients with stage I–II disease, the decision to pursue definitive radiotherapy rather than surgical resection was made by a multidisciplinary tumor board based on individual patient factors, such as advanced age, significant comorbidities, tumor location, or patient preference.

### Data collection and variable definitions

Demographic, clinical, laboratory, and treatment data were meticulously extracted from the hospital’s information system by two trained clinical researchers using a standardized data collection form. Any discrepancies were resolved through discussion with a senior oncologist. The collected data included age, gender, smoking and drinking history, TNM stage (according to the 8th edition of the AJCC Cancer Staging Manual), ECOG PS, and treatment details (e.g., concurrent chemotherapy). Baseline laboratory parameters, including neutrophil-to-lymphocyte ratio (NLR), systemic immune-inflammation index (SII), albumin, C-reactive protein (CRP), and lactate dehydrogenase (LDH), were recorded from tests performed within one week prior to the initiation of radiotherapy. The NLR was calculated as the absolute neutrophil count divided by the absolute lymphocyte count. The SII was calculated using the formula: SII = (platelet count × neutrophil count)/lymphocyte count. Body mass index (BMI) was calculated as weight in kilograms divided by the square of height in meters (kg/m^2^). Nutritional status was assessed using the Nutritional Risk Screening 2002 (NRS 2002) tool, with a score of ≥3 indicating nutritional risk. Nutritional risk was assessed using the validated NRS 2002 tool. The total score is the sum of three components: Nutritional Status Score (0–3 points): Based on weight loss, BMI, and recent food intake. Disease Severity Score (0–3 points): Based on the patient’s primary diagnosis and its metabolic demand. Age Adjustment: An additional point is added if the patient is 70 years or older. A total score of ≥3 points identifies patients “at nutritional risk” who would likely benefit from nutritional intervention. Body mass index (BMI) was calculated from height and weight measurements taken at the first clinical visit prior to treatment. Based on the World Health Organization classification, patients were stratified into two groups: a low BMI group (BMI < 18.5 kg/m^2^) and a non-low BMI group (BMI ≥ 18.5 kg/m^2^).

### Definition of variables and cut-off criteria

To ensure clarity and reproducibility, all key variables and their pre-defined cut-off values used for analysis are defined as follows. TNM Stage: Disease stage was classified according to the 8th edition of the American Joint Committee on Cancer (AJCC) Cancer Staging Manual for esophageal carcinoma (21). Performance status: The Eastern Cooperative Oncology Group Performance Status (ECOG PS) was assessed on a scale of 0 (fully active) to 5 (dead), as per the standard definition (22). Nutritional Risk Screening: Nutritional status was evaluated using the validated Nutritional Risk Screening 2002 (NRS 2002) tool. A score ≥3, which incorporates parameters of nutritional status, disease severity, and age, was used to define patients “at nutritional risk” requiring intervention, as per the tool’s validation (23). Body mass index (BMI): BMI was calculated as weight in kilograms divided by the square of height in meters (kg/m^2^). Patients were stratified into a low BMI group (BMI < 18.5 kg/m^2^) and a non-low BMI group (BMI ≥ 18.5 kg/m^2^) based on the World Health Organization (WHO) classification for underweight status in adults (24). Neutrophil-to-lymphocyte ratio (NLR): Calculated as the absolute neutrophil count divided by the absolute lymphocyte count from peripheral blood tests. An NLR ≥ 3 was defined as elevated, a cut-off consistently associated with worse survival in multiple gastrointestinal malignancies, including ESCC (25, 26). Systemic immune-inflammation index (SII): Calculated using the formula: Platelet count × Neutrophil count/Lymphocyte count. An SII ≥ 800 was considered high, based on its established prognostic value in esophageal cancer cohorts (27). Albumin: Serum albumin level was measured in g/L. Hypoalbuminemia was defined as albumin < 35 g/L, a common threshold indicative of nutritional depletion and a poor prognostic factor in cancer patients (28). C-reactive Protein (CRP): Serum CRP level was measured in mg/L. An elevated CRP level was defined as ≥ 5 mg/L, reflecting significant systemic inflammation (29). Lactate dehydrogenase (LDH): Serum LDH level was measured in U/L. An elevated LDH level was defined as > 250 U/L, a common upper limit of normal associated with high tumor burden and poor prognosis (30, 31).

### Radiotherapy and treatment protocols

All patients underwent computed tomography (CT)-based simulation for radiotherapy planning. The gross tumor volume (GTV) included the primary esophageal tumor and any involved regional lymph nodes (short-axis diameter ≥ 1 cm on CT or with pathological confirmation via endoscopic ultrasound or PET-CT avidity). The clinical target volume (CTV) encompassed the GTV with a 3 cm craniocaudal margin and a 0.5 cm radial margin to cover submucosal spread and microscopic disease. Elective nodal irradiation was performed for regional nodal stations according to the primary tumor’s location (e.g., supraclavicular fossa for cervical/upper thoracic tumors, celiac nodes for distal tumors). The planning target volume (PTV) was generated by adding a 0.5 cm isotropic margin to the CTV to account for setup variability and organ motion. This approach to target volume delineation is consistent with principles used in major clinical trials for definitive chemoradiation in esophageal cancer (3). All patients were treated with modern techniques, specifically intensity-modulated radiotherapy (IMRT) or volumetric modulated arc therapy (VMAT). The prescribed radiation dose was 50.4 Gy in 28 fractions (1.8 Gy per fraction) or 50–50.4 Gy in 25–28 fractions, aligning with standard definitive chemoradiation protocols. For patients receiving concurrent chemotherapy, the regimen consisted of carboplatin (AUC 2) and paclitaxel (50 mg/m^2^) administered weekly, a schedule commonly used in contemporary practice and derived from protocols like the CROSS trial (3). The decision to administer concurrent chemotherapy was made by a multidisciplinary tumor board based on the patient’s performance status, renal function, and comorbidities. During the study period (2021–2024), immune checkpoint inhibitors were not part of the standard definitive chemoradiation protocol for non-metastatic ESCC at our institution and were not administered to any patient in this cohort.

### Study outcomes and endpoint definitions

The primary endpoint of this study was overall survival (OS), defined as the time from the initiation of radiotherapy to death from any cause. The secondary endpoint was progression-free survival (PFS), defined as the time from the initiation of radiotherapy to the first occurrence of disease progression (locoregional recurrence or distant metastasis) or death from any cause, whichever occurred first. Disease progression was evaluated according to Response Evaluation Criteria in Solid Tumors (RECIST) version 1.1, based on imaging studies (CT, PET-CT) and/or endoscopic findings. Treatment-related adverse events (AEs) were graded throughout the treatment period and during follow-up visits according to the Common Terminology Criteria for Adverse Events (CTCAE) version 5.0.

### Statistical analysis

Categorical variables were presented as frequencies and percentages, and comparisons between the two BMI groups were performed using the Chi-square test or Fisher’s exact test, as appropriate. Continuous variables were expressed as mean ± standard deviation (SD) and compared using the Student’s *t*-test or the Mann–Whitney U test depending on the normality of the data distribution, which was assessed by the Shapiro–Wilk test. Survival curves for OS and PFS were generated using the Kaplan–Meier method, and differences between groups were compared with the log-rank test. To identify independent prognostic factors, univariate and multivariate analyses were conducted using Cox proportional hazards models. Variables with a *p*-value < 0.1 in the univariate analysis were included in the multivariate Cox regression model using a backward stepwise selection procedure. Subgroup analyses were pre-specified to assess the consistency of the BMI effect across various patient characteristics; interaction terms were tested using Cox models. All statistical tests were two-sided, and a *p*-value of less than 0.05 was considered statistically significant. Data analysis was performed using SPSS Statistics version 26.0 (IBM Corp., Armonk, NY, USA).

## Results

### Patient enrollment and study population

A total of 189 patients diagnosed with stage I–III esophageal squamous cell carcinoma and receiving definitive radiotherapy between January 1st 2021 and December 31th 2024 were initially screened for this real-world study. The patient selection process, detailed in the enrollment flowchart (Figure 1), involved the exclusion of 47 patients due to incomplete medical records, 2 patients with synchronous secondary malignancies, and 5 patients with an ECOG PS greater than 3. Consequently, 135 eligible patients constituted the final study cohort. Based on the World Health Organization classification for underweight status, the cohort was stratified into two groups: a low BMI group (BMI < 18.5 kg/m^2^, *n* = 33) and a non-low BMI group (BMI ≥ 18.5 kg/m^2^, *n* = 102).

### Baseline demographic and clinical characteristics

The baseline characteristics of the two groups are comprehensively summarized in Table 1. The distribution of age and sex was well-balanced between the groups. The vast majority of patients were aged 65 years or older (low BMI: 93.9% vs. non-low BMI: 93.1%, *p* = 1.000), and most were male (84.8% vs. 78.4%, *p* = 0.423). No significant differences were observed in smoking history (48.5% vs. 44.1%, *p* = 0.661) or drinking history (33.3% vs. 40.2%, *p* = 0.481).

**TABLE 1:** Baseline characteristics (*n* = 135).

Baseline characteristics	Low BMI group (*n* = 33)	Non–low BMI group (*n* = 102)	*P*
**Age, [years, *n* (%)]**			1.000
≥ 65	31 (93.9)	95 (93.1)	
< 65	2 (6.1)	7 (6.9)	
**Gender, *n* (%)**			0.423
Male	28 (84.8)	80 (78.4)	
Female	5 (15.2)	22 (21.6)	
**TNM stage, *n* (%)**			
I	4 (12.1)	26 (25.5)	0.011
II	3 (9.1)	26 (25.5)
III	26 (78.8)	50 (49)
**Smoking history, *n* (%)**			0.661
Yes	16 (48.5)	45 (44.1)	
No	17 (51.5)	57 (55.9)	
**Drinking history, *n* (%)**			0.481
Yes	11 (33.3)	41 (40.2)	
No	22 (66.7)	61 (59.8)	
**NRS2002, *n* (%)**			0.01
≥ 3	31 (93.9)	74 (72.5)	
< 3	2 (6.1)	28 (27.5)	
**ECOG PS, *n* (%)**			0.336
0–1	6 (18.2)	27 (26.5)	
2	27 (81.8)	75 (73.5)	
**Concurrent chemoradiotherapy, *n* (%)**			0.176
Yes	20 (60.6)	48 (47.1)	
No	13 (39.4)	54 (52.9)	
**Chemotherapy dose reduction, *n* (%)**			0.346
Yes	15 (45.5)	37 (36.3)	
No	18 (54.5)	65 (63.7)	
NLR, mean ± SD	4.41 ± 3.57	5.15 ± 7.98	0.607
SII, mean ± SD	873.12 ± 572.46	831.90 ± 1,315.97	0.456
Albumin (g/L), mean ± SD	37.71 ± 3.93	41.63 ± 9.13	0.212
CRP (mg/L), mean ± SD	11.78 ± 14.33	11.81 ± 20.51	0.869
LDH (U/L), mean ± SD	194.77 ± 40.95	198.65 ± 46.70	0.650

BMI, body mass index; NRS2002, Nutritional Risk Screening 2002; ECOG PS, Eastern Cooperative Oncology Group Performance Status; NLR, neutrophil-to-lymphocyte ratio; SII, systemic immune-inflammation index; CRP, C-reactive protein; LDH, lactate dehydrogenase.

However, notable disparities were identified in key clinical parameters. A significantly higher proportion of patients in the low BMI group presented with advanced TNM stage III disease (78.8%) compared to the non-low BMI group (49.0%). Correspondingly, the non-low BMI group had a higher prevalence of early-stage (I and II) disease (51.0% combined vs. 21.2%). Nutritional status, assessed by the NRS 2002 score, revealed a markedly higher prevalence of nutritional risk (score ≥ 3) in the Low BMI group (93.9% vs. 72.5%, *p* = 0.010). Furthermore, a non-significant trend toward lower serum albumin levels was observed in the low BMI group (37.71 ± 3.93 g/L vs. 41.63 ± 9.13 g/L, *p* = 0.212). Other parameters, including ECOG PS, rate of concurrent chemoradiotherapy and chemotherapy dose reduction, and systemic inflammation markers (NLR, SII, CRP, LDH), were comparable between the groups (all *p* > 0.05).

### Survival outcomes

**FIGURE 2:**
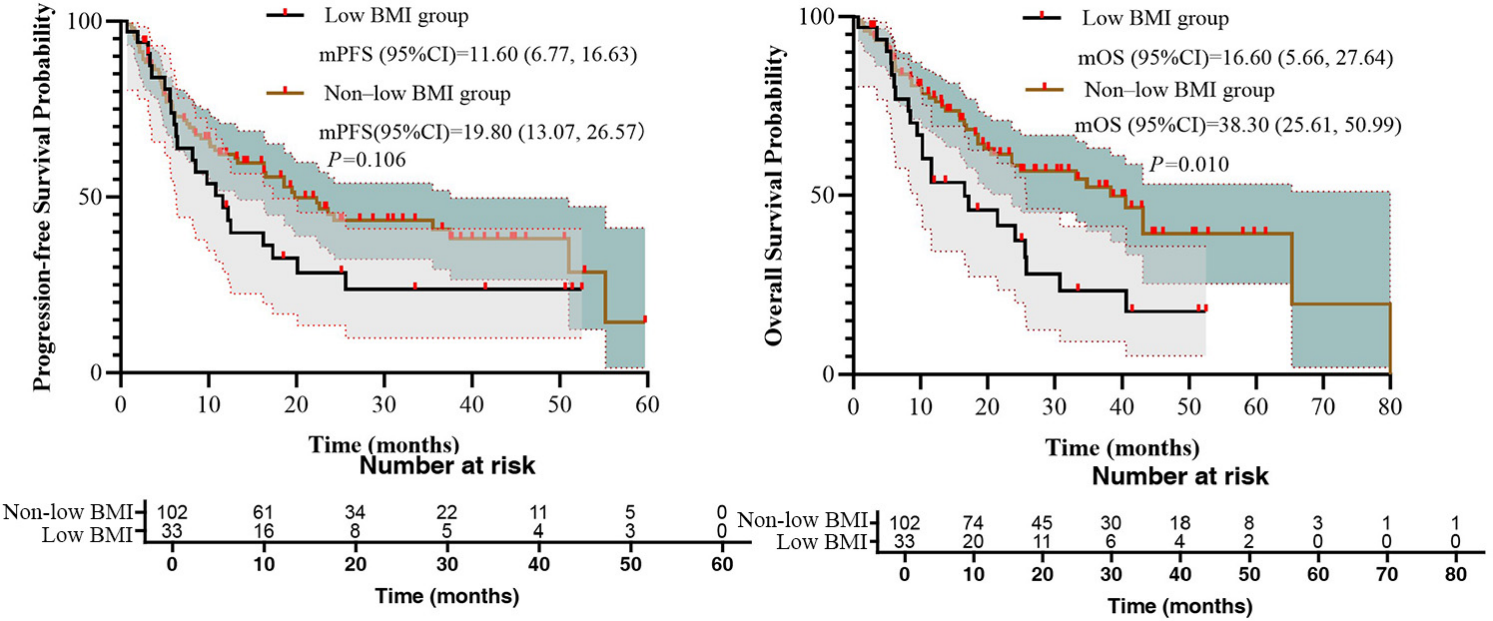
Kaplan–Meier curves for progression-free survival (PFS, left) overall survival (OS, right) between BMI groups. Comparison of PFS and OS in patients with low BMI (< 18.5 kg/m^2^) versus non-low BMI (≥ 18.5 kg/m^2^). The *p*-value was calculated using the log-rank test. BMI, body mass index.

With a median follow-up duration of 24.5 months (range: 5.2–43.1 months), Kaplan–Meier survival analysis was performed. The survival curves for PFS and OS are presented in Figure 2.

The analysis for PFS indicated a numerical disadvantage for the low BMI group, with a median PFS (mPFS) of 11.60 months (95% Confidence Interval [CI]: 6.77–16.63), compared to 19.80 months (95% CI: 13.07–26.57) in the non-low BMI group. However, the log-rank test determined that this difference was not statistically significant (*p* = 0.106). The study may have been underpowered to detect a difference in PFS due to the sample size limitation.

In contrast, the impact on overall survival was profound and statistically significant. Patients in the low BMI group had a markedly inferior median OS (mOS) of 16.60 months (95% CI: 5.66–27.64). Patients in the non-low BMI group achieved a significantly longer mOS of 38.30 months (95% CI: 25.61–50.99). The log-rank test confirmed the statistical significance of this near 22-month survival difference (*p* = 0.010).

### Subgroup analyses of survival

**FIGURE 3:**
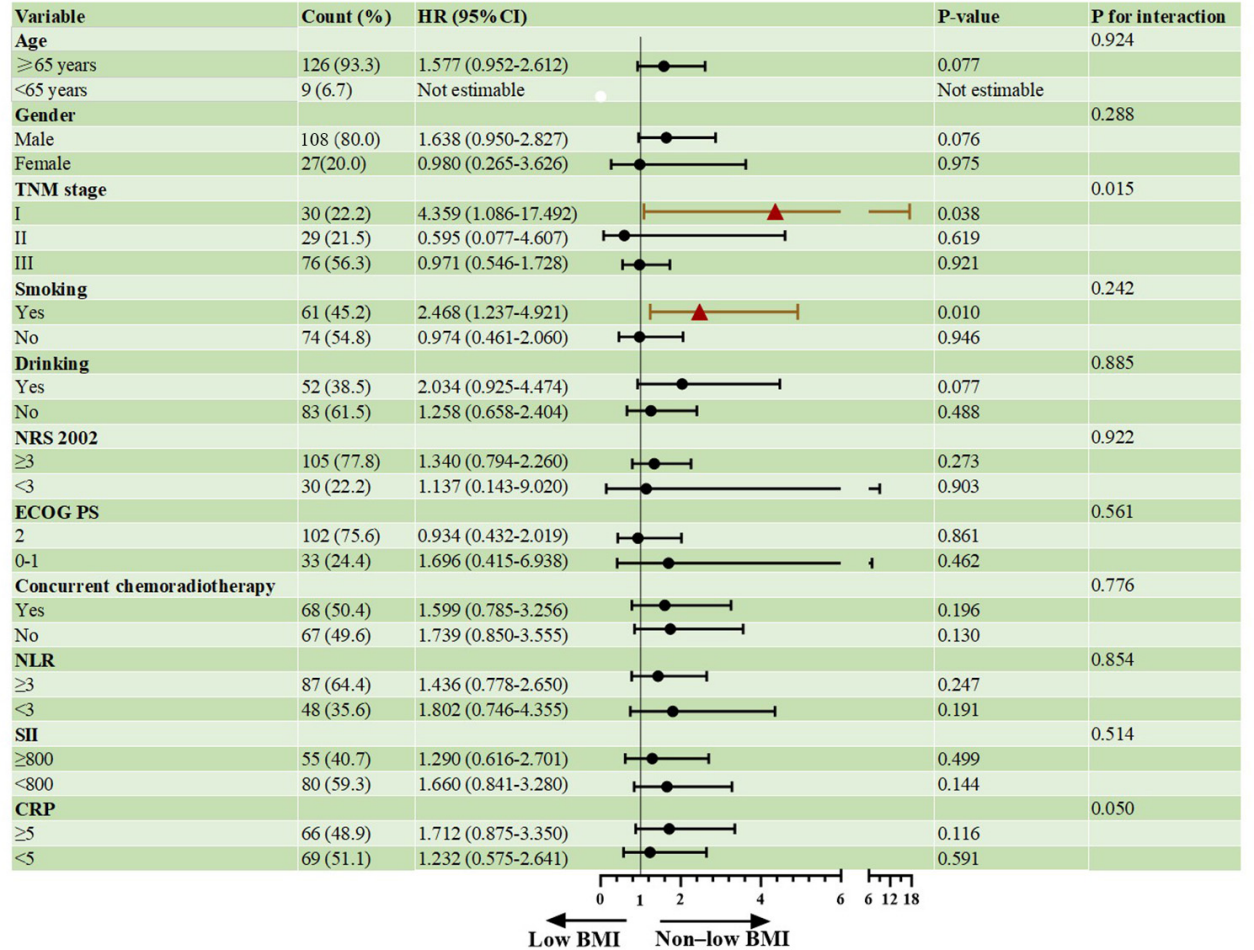
Forest plot of subgroup analyses for progression-free survival (PFS). Hazard ratios (HR) with 95% confidence intervals (CI) are shown. BMI, body mass index; NLR, neutrophil-to-lymphocyte ratio; SII, systemic immune-inflammation index; CRP, C-reactive protein; TNM, tumor-node-metastasis; ECOG PS, Eastern Cooperative Oncology Group Performance Status; CCRT, concurrent chemoradiotherapy. Nutritional Risk Screening 2002.

**FIGURE 4:**
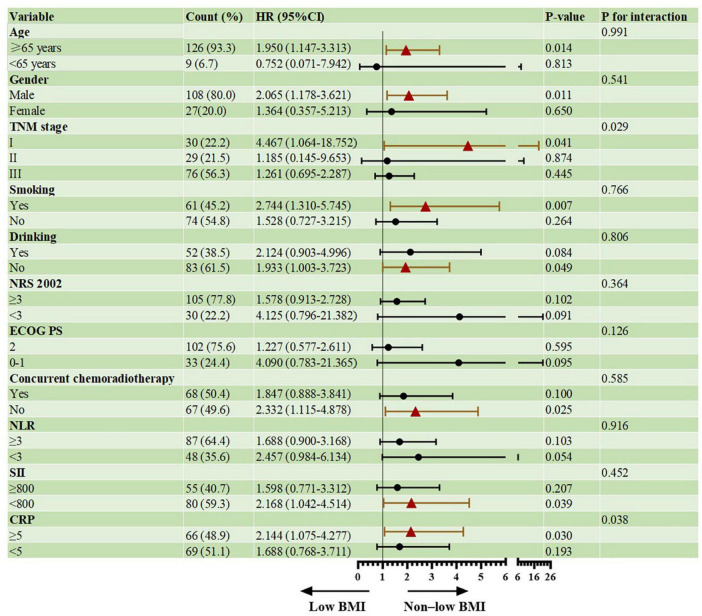
Forest plot of subgroup analyses for overall survival (OS). Hazard ratios (HR) with 95% confidence intervals (CI) are shown. BMI, body mass index; NLR, neutrophil-to-lymphocyte ratio; SII, systemic immune-inflammation index; CRP, C-reactive protein; TNM, tumor-node-metastasis; ECOG PS, Eastern Cooperative Oncology Group Performance Status; CCRT, concurrent chemoradiotherapy. Nutritional Risk Screening 2002.

To evaluate the consistency of the BMI effect across various patient demographics and clinical features, subgroup analyses were conducted for both PFS (Figure 3) and OS (Figure 4).

The PFS subgroup analysis (Figure 3) demonstrated that the detrimental effect of low BMI was particularly pronounced in several subgroups. These included patients with a smoking history (HR = 2. 468, 95% CI: 1.237–4.921, *p* = 0.01), and those with stage I group (HR = 4.359, 95% CI: 1.086–17.492, *p* = 0.038). A test for interaction revealed that the effect of low BMI on PFS differed significantly according to TNM stage (*p* for interaction = 0.015).

The OS subgroup analysis (Figure 4) revealed a similar interaction with TNM stage (*p* for interaction = 0.029). The adverse impact of low BMI was overwhelmingly concentrated in patients with stage I disease, where it was associated with a more than fourfold increased risk of mortality (HR = 4.467, 95% CI: 1.064–18.752, *p* = 0.041). Conversely, the effect was attenuated and non-significant in stages II and III. In addition, low BMI also significantly worsened OS in smokers (HR = 2.744, 95% CI: 1.310–5.745, *p* = 0.007), male gender (HR = 2.065, 95% CI: 1.178–3.621, *p* = 0.011), age more than 65 years old (HR = 1.950, 95% CI: 1.147–3.313, *p* = 0.014), no concurrent chemoradiotherapy (HR = 2.332, 95% CI: 1.115–4.878, *p* = 0.025), high SII (SII ≥ 800: HR = 2.168, 95% CI: 1.042–4.514, *p* = 0.039), and an elevated CRP (HR = 2.144, 95% CI: 1.075–4.277, *p* = 0.03).

### Univariate and multivariate cox regression analyses

**FIGURE 5:**
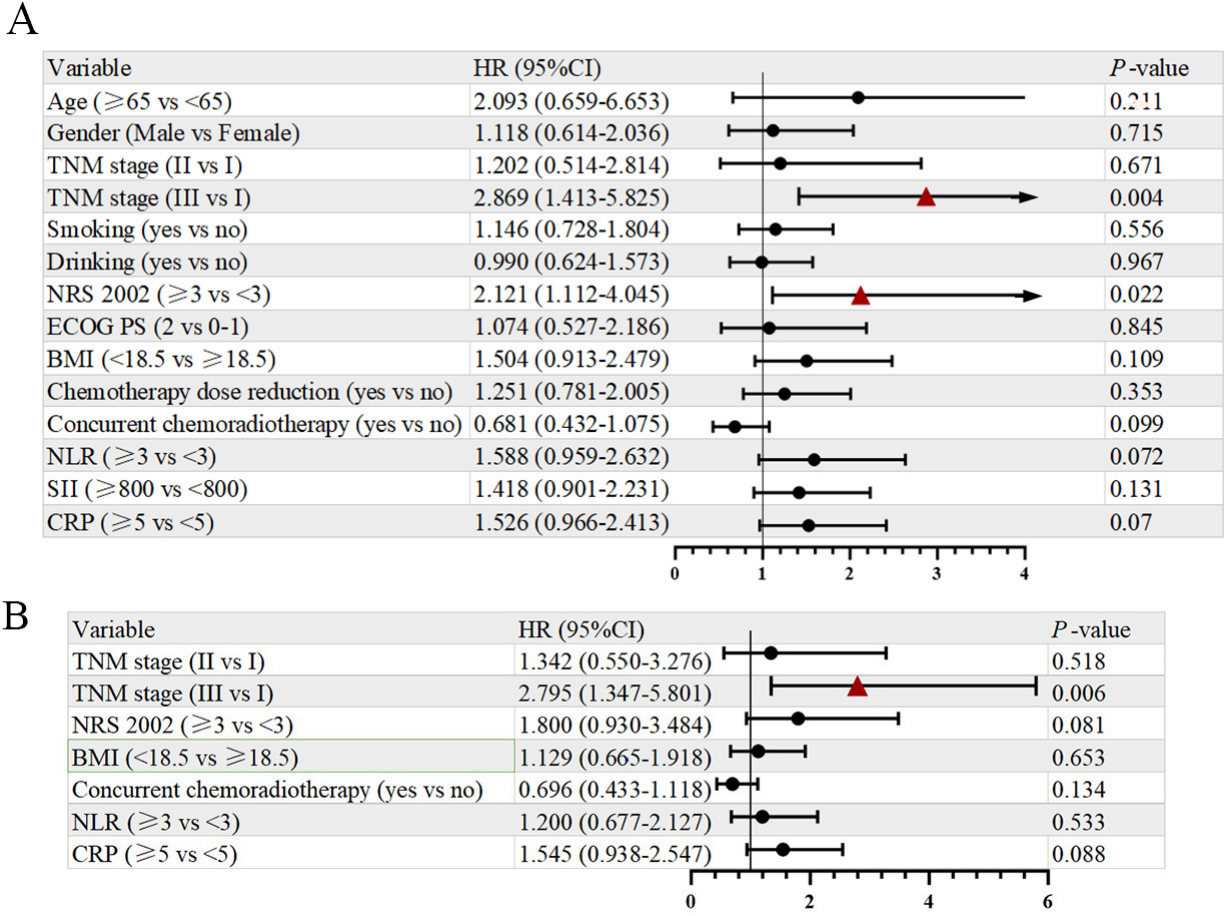
Univariate and multivariate Cox regression analysis for progression-free survival (PFS). **(A)** Univariate analysis. **(B)** Multivariate analysis. HR, hazard ratio; CI, confidence interval; NLR, neutrophil-to-lymphocyte ratio; SII, systemic immune-inflammation index; CRP, C-reactive protein; TNM, tumor-node-metastasis; ECOG PS, Eastern Cooperative Oncology Group Performance Status; CCRT, concurrent chemoradiotherapy. Nutritional Risk Screening 2002.

**FIGURE 6:**
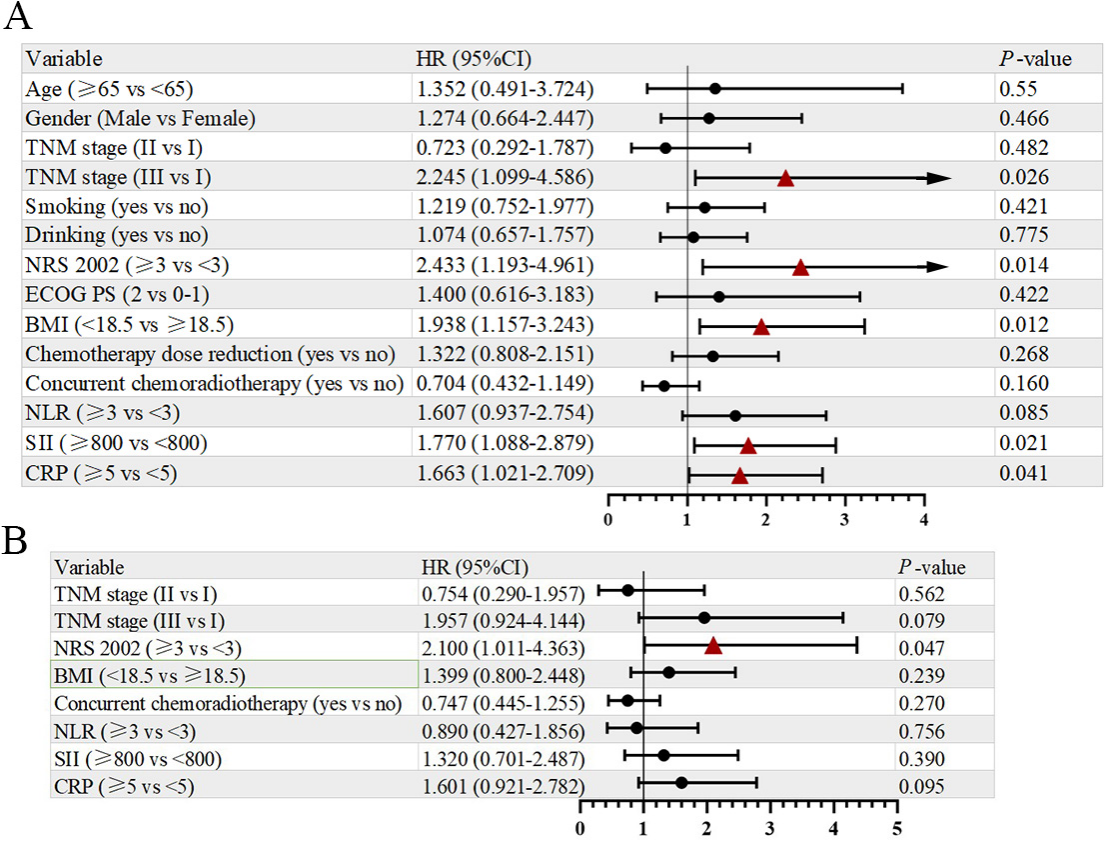
Univariate and multivariate Cox regression analysis for overall survival (OS). **(A)** Univariate analysis. **(B)** Multivariate analysis. HR, hazard ratio; CI, confidence interval; NLR, neutrophil-to-lymphocyte ratio; SII, systemic immune-inflammation index; CRP, C-reactive protein; TNM, tumor-node-metastasis; ECOG PS, Eastern Cooperative Oncology Group Performance Status; CCRT, concurrent chemoradiotherapy. Nutritional Risk Screening 2002.

Forest plots from the univariate and multivariate Cox proportional hazards models are shown in Figure 5 (for PFS) and Figure 6 (for OS).

In the univariate analysis for PFS (Figure 5A), several factors predicted poorer outcomes: advanced TNM stage (III vs. I: HR = 2.869, 95% CI: 1.413–5.825, *p* = 0.004), and presence of nutritional risk (NRS2002 ≥ 3: HR = 2.121, 95% CI: 1.112–4.045, *p* = 0.022). The multivariate analysis for PFS (Figure 5B), which adjusted for these factors, confirmed that advanced TNM stage (III vs. I: HR = 2.795, 95% CI: 1.347–5.801, *p* = 0.006) was an independent prognostic factor.

For OS (Figure 6A), univariate analysis identified advanced TNM stage (III vs. I: HR = 2.245, 95% CI: 1.099–4.586, *p* = 0.026), nutritional risk (NRS2002 ≥ 3: HR = 2.433, 95% CI: 1.193–4.961, *p* = 0.014), low BMI (BMI < 18.5: HR = 1.938, 95% CI: 1.157–3.243, *p* = 0.012), CRP (CRP ≥ 5: HR = 1.663, 95% CI: 1.021–2.709, *p* = 0.041), and high SII (SII ≥ 800: HR = 1.770, 95% CI: 1.088–2.879, *p* = 0.021) as significant predictors. In the multivariate model for OS (Figure 6B), nutritional risk (NRS2002 ≥ 3: HR = 2.100, 95% CI: 1.011–4.363, *p* = 0.047) remained an independent predictor of inferior survival.

### Treatment-related adverse events

The frequencies of treatment-related adverse events for both the low BMI and non-low BMI groups are detailed in Table 2. Overall, adverse events were numerically more frequent in the low BMI group across several toxicity categories, including anemia, thrombocytopenia, neutropenia, diarrhea, fatigue, and radiation esophagitis. For severe toxicities, the low BMI group also showed numerically higher rates of several grade 3–4 events, including anemia, neutropenia, thrombocytopenia, and radiation esophagitis. Given the small number of severe events in each category, Fisher’s exact test was used to compare individual grade 3–4 toxicities between groups. None of the individual grade 3–4 toxicity comparisons reached statistical significance. Specifically, grade 3–4 anemia occurred in 12.1% versus 2.9% of patients (*p* = 0.060), grade 3–4 neutropenia in 12.1% versus 2.9% (*p* = 0.060), thrombocytopenia in 9.1% versus 2.0% (*p* = 0.094), and radiation esophagitis in 15.1% versus 5.9% (*p* = 0.136) in the low BMI and non-low BMI groups, respectively. The full results of Fisher’s exact tests for grade 3–4 toxicities are provided in Supplementary Table 1.

**TABLE 2:** Adverse events.

Adverse events	Low BMI group (*n* = 33)		Non–low BMI group (*n* = 102)	
	Grade 1–2, *n* (%)	Grade 3–4, *n* (%)	Grade 1–2, *n* (%)	Grade 3–4, *n* (%)
Diarrhea	10 (30.3)	2 (6.1)	15 (14.7)	1 (1.0)
Anemia	22 (66.7)	4 (12.1)	45 (44.1)	3 (2.9)
Thrombocytopenia	14 (42.4)	3 (9.1)	25 (24.5)	2 (2.0)
Elevated creatinine	6 (18.2)	1 (3.0)	10 (9.8)	1 (1.0)
Fatigue	27 (81.8)	3 (9.1)	65 (63.7)	4 (3.9)
Nausea	8 (24.2)	2 (6.1)	18 (17.6)	2 (2.0)
Vomiting	7 (21.2)	1 (3.0)	12 (11.8)	1 (1.0)
Neutropenia	14 (42.4)	4 (12.1)	20 (19.6)	3 (2.9)
Radiation esophagitis	20 (60.6)	5 (15.1)	45 (44.1)	6 (5.9)
Radiation pneumonia	6 (18.2)	2 (6.1)	10 (9.8)	2 (2.0)

## Discussion

This real-world study provides a comprehensive analysis of the impact of low BMI on clinical outcomes in a well-defined cohort of 135 patients with stage I–III ESCC treated with definitive radiotherapy. Our principal findings demonstrate that patients with a low BMI (< 18.5 kg/m^2^) experienced significantly inferior overall survival compared to those with a non-low BMI, despite no statistically significant difference in PFS. Furthermore, low BMI was associated with a numerically higher burden of treatment-related toxicities. These results underscore the critical prognostic value of nutritional status, as simply measured by BMI, in the management of ESCC patients undergoing radical radiotherapy.

The most striking finding of our study is the profound disparity in OS, with a near 22-month difference in median survival (16.60 vs. 38.30 months, *p* = 0.010) favoring the non-low BMI group. This aligns with the established literature linking malnutrition and cancer cachexia to poor survival across various malignancies (32). However, the lack of a significant difference in PFS (11.60 vs. 19.80 months, *p* = 0.106) presents a nuanced picture. This dissociation between PFS and OS suggests that the survival disadvantage in low BMI patients may not be solely driven by accelerated tumor progression. While the retrospective nature of our study precludes a formal analysis of specific causes of death, several lines of evidence support this interpretation. First, the negative prognostic impact of low BMI remained significant after multivariate adjustment for TNM stage and treatment modality, suggesting its effect extends beyond being a mere correlate of advanced disease, a primary driver of cancer-specific mortality. Second, the observed dissociation between OS and PFS is conceptually consistent with a competing risks framework, where an elevated risk of non-cancer death (e.g., from treatment complications in a vulnerable host) competes with the risk of death from progression. Finally, the significantly higher rate of severe toxicities in the low BMI group provides a plausible clinical pathway linking poor baseline status to increased mortality from treatment-related morbidity. Consequently, the survival disadvantage in low BMI patients is likely multifactorial, driven by both compromised treatment tolerance and potentially aggravated by the underlying frailty that low BMI signifies. Rather, this pattern may reflect the broader clinical vulnerability represented by low BMI, including limited physiological reserve and reduced tolerance to intensive oncologic treatment, rather than accelerated tumor progression alone. This interpretation is consistent with the observed toxicity profile, in which hematological and non-hematological adverse events tended to be more frequent in the low BMI group. The increased incidence of severe (Grade 3–4) anemia, neutropenia, and thrombocytopenia highlights a reduced bone marrow tolerance, while the higher rates of radiation esophagitis and pneumonia suggest diminished tissue resilience (33). This creates a vicious cycle where treatment intolerance leads to dose reductions or interruptions, potentially compromising tumor control, and/or directly contributing to mortality from complications.

The numerically higher toxicity burden and inferior survival observed in low BMI patients necessitate a paradigm shift toward proactive and integrated nutritional management. Our findings strongly advocate for the systematic implementation of routine nutritional screening as a mandatory component of the initial workup for ESCC patients planned for definitive radiotherapy. Simple and validated tools like the Nutritional Risk Screening 2002 (NRS 2002) or the Patient-Generated Subjective Global Assessment (PG-SGA) are efficient for identifying patients at risk (23, 34). For a more precise evaluation of body composition, which may be a stronger prognostic factor than BMI alone, computed tomography (CT)-based analysis of skeletal muscle mass (sarcopenia) and adipose tissue should be considered, especially in research settings or when available (35, 36). For patients identified as at-risk or malnourished, early and multimodal nutritional intervention must be initiated prior to and sustained throughout treatment. Evidence from randomized controlled trials in other radiotherapy-sensitive cancers supports this approach. For instance, in head and neck cancer patients undergoing radiotherapy, individualized dietary counseling and oral nutritional supplements have been shown to improve nutritional intake, reduce treatment-related weight loss, decrease the incidence and severity of toxicities like mucositis, and better preserve quality of life (37, 38). The intervention should be tailored to the patient’s ability to swallow and may range from intensive dietary counseling and high-protein, high-calorie oral nutritional supplements, to, in cases of severe dysphagia, the timely placement of a nasogastric tube or percutaneous endoscopic gastrostomy (PEG) for enteral feeding (39). While our study was not designed to evaluate such interventions, the stark outcome disparity we observed provides a compelling rationale for future prospective trials to test whether protocolized, early nutritional support can abrogate the survival gap and reduce toxicity in this vulnerable ESCC subgroup.

Our multivariate Cox regression analyses further clarified the independent prognostic factors. For OS, nutritional risk (NRS2002 ≥ 3: HR = 2.100, *p* = 0.047) remained a significant independent predictor, while the binary low BMI variable did not retain significance. This is likely because the NRS 2002 score, a composite measure incorporating BMI, weight loss, and disease severity, captured the prognostic information related to undernutrition more comprehensively. The very high prevalence of nutritional risk in the low BMI group (93.9%) supports this interpretation. Therefore, while low BMI (< 18.5 kg/m^2^) was strongly associated with inferior OS in univariate analysis, its prognostic impact appears to be mediated through the broader construct of nutritional risk, which includes but is not limited to low body mass.

The subgroup analyses provided exploratory observations, particularly the interaction between BMI and TNM stage for both PFS and OS. The association between low BMI and adverse outcomes appeared more evident in patients with stage I disease. For instance, in stage I patients, low BMI was associated with a more than fourfold increased risk of mortality (HR = 4.467 for OS). However, this subgroup contained only 4 patients with low BMI, resulting in a very wide confidence interval (1.064–18.752). Therefore, this observation should be interpreted with extreme caution and should be considered hypothesis-generating only; it underscores the need for validation in larger, prospective cohorts rather than supporting a definitive conclusion. Nevertheless, the pattern observed in our data raises the possibility that host-related vulnerability may contribute to survival differences, particularly when tumor-related risk is less dominant. This interpretation remains speculative because of the very small subgroup size and multiple subgroup comparisons, and no firm clinical conclusion can be drawn regarding a stage-specific effect of low BMI. One plausible explanation is that in patients with early-stage disease, where the tumor burden is smaller, host-related factors like nutrition and systemic inflammation may play a relatively more dominant role in determining outcomes compared to patients with advanced disease, where the aggressive biology of the tumor itself becomes the primary driver of mortality (35). In stage III patients, the overpowering effect of the high tumor burden may mask the subtler impact of nutritional status. This possible interaction warrants further investigation in larger cohorts before any stage-specific nutritional intervention strategy can be proposed.

The relationship between low BMI and systemic inflammation is complex. While baseline NLR, SII, and CRP levels were comparable between groups, the subgroup analysis indicated that the negative impact of low BMI was more pronounced in patients with elevated CRP. This suggests a potential synergy between nutritional depletion and a pro-inflammatory state, both hallmarks of cancer cachexia (40). The systemic inflammation associated with cachexia can promote muscle wasting, suppress appetite, and contribute to treatment resistance, creating a feed-forward loop that exacerbates the patient’s decline (12). The association between SII and survival also warrants cautious interpretation. Although high SII was associated with worse OS in univariate analysis, it did not remain significant in the multivariate model, suggesting that its prognostic value may be influenced by other clinical and nutritional factors (41).

The numerically higher toxicity burden observed in low BMI patients has direct clinical implications. It underscores the imperative for proactive and intensive supportive care in this vulnerable population. Our findings strongly argue for the integration of routine nutritional screening and early nutritional intervention as an integral part of the multidisciplinary management for ESCC patients undergoing radiotherapy. Randomized trials have demonstrated that targeted nutritional support can reduce treatment-related toxicities and improve quality of life in cancer patients (38, 42). While our study was not designed to evaluate interventions, the stark contrast in toxicity profiles suggests that simply identifying low BMI patients and implementing standardized nutritional protocols, such as dietary counseling, oral nutritional supplements, or, in severe cases, enteral nutrition, could potentially mitigate adverse events and improve treatment tolerance and completion rates.

Several limitations of our study must be acknowledged. First, its retrospective and single-center nature inherits the potential for selection bias and unmeasured confounding factors. While we adjusted for key clinical variables, residual confounding may persist. Second, BMI is a crude measure of nutritional status and does not differentiate between loss of fat mass and skeletal muscle mass (sarcopenia). The use of computed tomography (CT)-based body composition analysis would provide a more precise assessment of sarcopenia, which might be an even stronger predictor of outcomes than BMI alone (36, 43–45). Third, the sample size, particularly of the low BMI group (*n* = 33), limits the statistical power for some subgroup analyses and multivariate models. Although exploratory Fisher’s exact tests were performed for individual grade 3–4 toxicities, the analysis was limited by the small number of severe events and multiplicity of comparisons. Finally, as a real-world study, treatment details, including chemotherapy regimens and radiotherapy dose/fractionation, although generally standardized, may have had minor variations that were not fully accounted for.

In conclusion, low baseline BMI was associated with poorer unadjusted overall survival and a numerically higher burden of treatment-related toxicities in patients with ESCC receiving definitive radiotherapy. However, after accounting for TNM stage and NRS2002-defined nutritional risk, BMI did not remain independently associated with OS or PFS. These findings indicate that BMI functions primarily as a practical and readily available surrogate marker of nutritional vulnerability and adverse baseline clinical status, rather than as an independent prognostic factor. The observed dissociation between PFS and OS, together with the exploratory stage-specific findings, suggests that host-related factors and treatment tolerance may contribute to survival outcomes, but these interpretations should be considered hypothesis-generating given the retrospective design and limited subgroup sample size. Overall, our results support the clinical value of comprehensive nutritional assessment beyond BMI alone and highlight the need for prospective studies to determine whether protocolized nutritional screening and early supportive intervention can improve treatment tolerance and clinical outcomes in this vulnerable patient population.

## Data Availability

The raw data supporting the conclusions of this article will be made available by the authors, without undue reservation.

## References

[1] Sung H Ferlay J Siegel RL Laversanne M Soerjomataram I Jemal A et al. Global cancer statistics 2020: GLOBOCAN estimates of incidence and mortality worldwide for 36 cancers in 185 countries. *CA Cancer J Clin*. (2021) 71:209–49. 10.3322/caac.21660 33538338

[2] Abnet CC Arnold M Wei WQ. Epidemiology of esophageal squamous cell carcinoma. *Gastroenterology*. (2018) 154:360–73. 10.1053/j.gastro.2017.08.023 28823862 PMC5836473

[3] van Hagen P Hulshof MC van Lanschot JJ Steyerberg EW van Berge Henegouwen MI Wijnhoven BP et al. Preoperative chemoradiotherapy for esophageal or junctional cancer. *N Engl J Med*. (2012) 366:2074–84. 10.1056/NEJMoa1112088 22646630

[4] Shapiro J van Lanschot JJB Hulshof MCCM van Hagen P van Berge Henegouwen MI Wijnhoven BPL et al. Neoadjuvant chemoradiotherapy plus surgery versus surgery alone for oesophageal or junctional cancer (CROSS): long-term results of a randomised controlled trial. *Lancet Oncol*. (2015) 16:1090–8. 10.1016/S1470-2045(15)00040-6 26254683

[5] Garant A Whitaker TJ Spears GM Routman DM Harmsen WS Wilhite TJ et al. A comparison of patient-reported health-related quality of life during proton versus photon chemoradiation therapy for esophageal cancer. *Pract Radiat Oncol*. (2019) 9:410–7. 10.1016/j.prro.2019.07.003 31310815

[6] Yamashita H Omori M Okuma K Kobayashi R Igaki H Nakagawa K. Longitudinal assessments of quality of life and late toxicities before and after definitive chemoradiation for esophageal cancer. *Jpn J Clin Oncol*. (2014) 44:78–84. 10.1093/jjco/hyt170 24220801

[7] Semenkovich TR Meyers BF. Understanding the impact of neoadjuvant chemoradiation on health-related quality of life in esophageal cancer. *Video Assist Thorac Surg*. (2018) 3:26. 10.21037/vats.2018.06.01 30221248 PMC6135084

[8] Zhou XL Yu CH Wang WW Ji FZ Xiong YZ Zhu WG et al. Concurrent chemoradiotherapy with S-1 compared with concurrent chemoradiotherapy with docetaxel and cisplatin for locally advanced esophageal squamous cell carcinoma. *Radiat Oncol*. (2021) 16:94. 10.1186/s13014-021-01821-6 34039375 PMC8157673

[9] Martin L Senesse P Gioulbasanis I Antoun S Bozzetti F Deans C et al. Diagnostic criteria for the classification of cancer-associated weight loss. *J Clin Oncol*. (2015) 33:90–9. 10.1200/JCO.2014.56.1894 25422490

[10] Calle EE Kaaks R. Overweight, obesity and cancer: epidemiological evidence and proposed mechanisms. *Nat Rev Cancer*. (2004) 4:579–91. 10.1038/nrc1408 15286738

[11] Fearon K Strasser F Anker SD Bosaeus I Bruera E Fainsinger RL et al. Definition and classification of cancer cachexia: an international consensus. *Lancet Oncol*. (2011) 12:489–95. 10.1016/S1470-2045(10)70218-7 21296615

[12] Argilés JM Busquets S Stemmler B López-Soriano FJ. Cancer cachexia: understanding the molecular basis. *Nat Rev Cancer*. (2014) 14:754–62. 10.1038/nrc3829 25291291

[13] Mantovani G Macciò A Madeddu C Serpe R Massa E Dessì M et al. Randomized phase III clinical trial of five different arms of treatment in 332 patients with cancer cachexia. *Oncologist*. (2010) 15:200–11. 10.1634/theoncologist.2009-0153 20156909 PMC3227938

[14] Fukuda Y Yamamoto K Hirao M Nishikawa K Maeda S Haraguchi N et al. Prevalence of malnutrition among gastric cancer patients undergoing gastrectomy and optimal preoperative nutritional support for preventing surgical site infections. *Ann Surg Oncol*. (2015) 22:S778–85. 10.1245/s10434-015-4820-9 26286199

[15] Lee S Jang J Abe SK Rahman S Saito E Islam R et al. Association between body mass index and oesophageal cancer mortality: a pooled analysis of prospective cohort studies with >800 000 individuals in the Asia Cohort Consortium. *Int J Epidemiol*. (2022) 51:1190–203. 10.1093/ije/dyac023 35229874 PMC9365631

[16] Deng HY Qin CL Qiu XM Zhou Q. Does high body mass index have any impact on survival of patients undergoing oesophagectomy for oesophageal cancer? *Interact Cardiovasc Thorac Surg*. (2018) 26:693–5. 10.1093/icvts/ivx403 29253176

[17] Yip C Goh V Davies A Gossage J Mitchell-Hay R Hynes O et al. Assessment of sarcopenia and changes in body composition after neoadjuvant chemotherapy and associations with clinical outcomes in oesophageal cancer. *Eur Radiol*. (2014) 24:998–1005. 10.1007/s00330-014-3110-4 24535076

[18] Urrizola A Dajani O Aass N Bjerkeset E Hjermstad MJ Kaasa S et al. Nutrition impact symptom monitoring and weight loss outcomes: a longitudinal radiotherapy study. *BMJ Support Palliat Care*. (2025) 15:522–5. 10.1136/spcare-2024-004939 38862183

[19] Jin S Lu Q Sun Y Xiao S Zheng B Pang D et al. Nutrition impact symptoms and weight loss in head and neck cancer during radiotherapy: a longitudinal study. *BMJ Support Palliat Care*. (2021) 11:17–24. 10.1136/bmjspcare-2019-002077 32019753

[20] Isenring EA Bauer JD Capra S. Nutrition support using the American Dietetic Association medical nutrition therapy protocol for radiation oncology patients improves dietary intake compared with standard practice. *J Am Diet Assoc*. (2007) 107:404–12. 10.1016/j.jada.2006.12.007 17324657

[21] Chen M Li X Chen Y Liu P Chen Z Shen M et al. Proposed revision of the 8th edition AJCC clinical staging system for esophageal squamous cell cancer treated with definitive chemo-IMRT based on CT imaging. *Radiat Oncol*. (2019) 14:54. 10.1186/s13014-019-1258-4 30922343 PMC6437982

[22] Oken MM Creech RH Tormey DC Horton J Davis TE McFadden ET et al. Toxicity and response criteria of the Eastern Cooperative Oncology Group. *Am J Clin Oncol.* (1982) 5:649–55. 7165009

[23] Kondrup J Rasmussen HH Hamberg O Stanga Z. Nutritional risk screening (NRS 2002): a new method based on an analysis of controlled clinical trials. *Clin Nutr*. (2003) 22:321–36. 10.1016/s0261-5614(02)00214-5 12765673

[24] Who. Physical status: the use and interpretation of anthropometry. Report of a WHO Expert Committee. *World Health Organ Tech Rep Ser.* (1995) 854:1–452. 8594834

[25] He W Yin C Guo G Jiang C Wang F Qiu H et al. Initial neutrophil lymphocyte ratio is superior to platelet lymphocyte ratio as an adverse prognostic and predictive factor in metastatic colorectal cancer. *Med Oncol*. (2013) 30:439. 10.1007/s12032-012-0439-x 23307251

[26] Hoshino S Takeuchi M Kawakubo H Kobayashi R Matsuda S Irino T et al. Neutrophil-to-lymphocyte ratio change predicts histological response to and oncological outcome of neoadjuvant chemotherapy for esophageal squamous cell carcinoma. *Esophagus*. (2022) 19:426–35. 10.1007/s10388-021-00901-6 35059908

[27] Hu B Yang XR Xu Y Sun YF Sun C Guo W et al. Systemic immune-inflammation index predicts prognosis of patients after curative resection for hepatocellular carcinoma. *Clin Cancer Res*. (2014) 20:6212–22. 10.1158/1078-0432.CCR-14-0442 25271081

[28] Arrieta O Michel Ortega RM Villanueva-Rodríguez G Serna-Thomé MG Flores-Estrada D Diaz-Romero C et al. Association of nutritional status and serum albumin levels with development of toxicity in patients with advanced non-small cell lung cancer treated with paclitaxel-cisplatin chemotherapy: a prospective study. *BMC Cancer*. (2010) 10:50. 10.1186/1471-2407-10-50 20170547 PMC2843671

[29] Lehtomäki K Mustonen H Kellokumpu-Lehtinen PL Joensuu H Hermunen K Soveri LM et al. Lead time and prognostic role of serum CEA, CA19-9, IL-6, CRP, and YKL-40 after adjuvant chemotherapy in colorectal cancer. *Cancers (Basel)*. (2021) 13:3892. 10.3390/cancers13153892 34359796 PMC8345682

[30] Chen Q Li GL Zhu HQ Yu JD Chen ZP Wu JY et al. The neutrophil-to-lymphocyte ratio and lactate dehydrogenase combined in predicting liver metastasis and prognosis of colorectal cancer. *Front Med (Lausanne)*. (2023) 10:1205897. 10.3389/fmed.2023.1205897 37425297 PMC10326518

[31] Cheng M Li G Liu Z Yang Q Jiang Y. Pretreatment neutrophil-to-lymphocyte ratio and lactate dehydrogenase predict the prognosis of metastatic cervical cancer treated with combination immunotherapy. *J Oncol*. (2022) 2022:1828473. 10.1155/2022/1828473 36304986 PMC9596258

[32] Arends J Bachmann P Baracos V Barthelemy N Bertz H Bozzetti F et al. ESPEN guidelines on nutrition in cancer patients. *Clin Nutr*. (2017) 36:11–48. 10.1016/j.clnu.2016.07.015 27637832

[33] Hill A Kiss N Hodgson B Crowe TC Walsh AD. Associations between nutritional status, weight loss, radiotherapy treatment toxicity and treatment outcomes in gastrointestinal cancer patients. *Clin Nutr*. (2011) 30:92–8. 10.1016/j.clnu.2010.07.015 20719409

[34] Jager-Wittenaar H Ottery FD. Assessing nutritional status in cancer: role of the patient-generated subjective global assessment. *Curr Opin Clin Nutr Metab Care*. (2017) 20:322–9. 10.1097/MCO.0000000000000389 28562490

[35] Martin L Birdsell L Macdonald N Reiman T Clandinin MT McCargar LJ et al. Cancer cachexia in the age of obesity: skeletal muscle depletion is a powerful prognostic factor, independent of body mass index. *J Clin Oncol*. (2013) 31:1539–47. 10.1200/JCO.2012.45.2722 23530101

[36] Prado CM Lieffers JR McCargar LJ Reiman T Sawyer MB Martin L et al. Prevalence and clinical implications of sarcopenic obesity in patients with solid tumours of the respiratory and gastrointestinal tracts: a population-based study. *Lancet Oncol*. (2008) 9:629–35. 10.1016/S1470-2045(08)70153-0 18539529

[37] Ravasco P Monteiro-Grillo I Vidal PM Camilo ME. Dietary counseling improves patient outcomes: a prospective, randomized, controlled trial in colorectal cancer patients undergoing radiotherapy. *J Clin Oncol*. (2005) 23:1431–8. 10.1200/JCO.2005.02.054 15684319

[38] Ravasco P Monteiro-Grillo I Marques Vidal P Camilo ME. Impact of nutrition on outcome: a prospective randomized controlled trial in patients with head and neck cancer undergoing radiotherapy. *Head Neck*. (2005) 27:659–68. 10.1002/hed.20221 15920748

[39] Bozzetti F Arends J Lundholm K Micklewright A Zurcher G Muscaritoli M et al. Guidelines on parenteral nutrition: non-surgical oncology. *Clin Nutr*. (2009) 28:445–54. 10.1016/j.clnu.2009.04.011 19477052

[40] Argilés JM Busquets S Toledo M López-Soriano FJ. The role of cytokines in cancer cachexia. *Curr Opin Support Palliat Care*. (2009) 3:263–8. 10.1097/SPC.0b013e3283311d09 19713854

[41] Al Murri AM Wilson C Lannigan A Doughty JC Angerson WJ McArdle CS et al. Evaluation of the relationship between the systemic inflammatory response and cancer-specific survival in patients with primary operable breast cancer. *Br J Cancer*. (2007) 96:891–5. 10.1038/sj.bjc.6603682 17375036 PMC2360103

[42] Bozzetti F. *Nutrition*al supplementation in advanced cancer patients: Re: “Influence of a nutritional intervention on dietary intake and quality of life in cancer patients”. *Nutrition.* (2014) 30:957–8. 10.1016/j.nut.2013.12.018 24985018

[43] Wang J Gao Q Chen J. Efficacy and safety of immune checkpoint inhibitor rechallenge therapy in the treatment of advanced acquired immune-resistant non-small cell lung cancer: a retrospective study. *Front Oncol*. (2025) 15:1621860. 10.3389/fonc.2025.1621860 41059304 PMC12497850

[44] Fang R Yan L Xu S Xu Y Gan T Gong J et al. Unraveling the obesity paradox in small cell lung cancer immunotherapy: unveiling prognostic insights through body composition analysis. *Front Immunol*. (2024) 15:1439877. 10.3389/fimmu.2024.1439877 39253074 PMC11381398

[45] Horibe M Takahashi N Weston AD Philbrick K Yamamoto S Takahashi H et al. Association between computerized tomography (CT) study of body composition and severity of acute pancreatitis: use of a novel Z-score supports obesity paradox. *Clin Nutr*. (2022) 41:1676–9. 10.1016/j.clnu.2022.06.010 35777106

