# Supplementary material for: Prognostic impact of low BMI on outcomes following definitive radiotherapy for esophageal squamous cell carcinoma
Source: Front Nutr. 2026 Jun 17;13:1774890. doi: 10.3389/fnut.2026.1774890 (PMC13318609; doi:10.3389/fnut.2026.1774890)
Supplement: Supplementary file 1 [file Table_1.docx]

**Supplementary Table 1. Fisher's exact tests for grade 3–4 treatment-related adverse events**

| **Adverse event** | **Low BMI group (n=33), n (%)** | **Non-low BMI group (n=102), n (%)** | **P-value** |
| --- | --- | --- | --- |
| Diarrhea | 2 (6.1) | 1 (1.0) | 0.148 |
| Anemia | 4 (12.1) | 3 (2.9) | 0.060 |
| Thrombocytopenia | 3 (9.1) | 2 (2.0) | 0.094 |
| Elevated creatinine | 1 (3.0) | 1 (1.0) | 0.431 |
| Fatigue | 3 (9.1) | 4 (3.9) | 0.361 |
| Nausea | 2 (6.1) | 2 (2.0) | 0.251 |
| Vomiting | 1 (3.0) | 1 (1.0) | 0.431 |
| Neutropenia | 4 (12.1) | 3 (2.9) | 0.060 |
| Radiation esophagitis | 5 (15.1) | 6 (5.9) | 0.136 |
| Radiation pneumonia | 2 (6.1) | 2 (2.0) | 0.251 |
